# Discovery and analysis of consistent active sub-networks in cancers

**DOI:** 10.1186/1471-2105-14-S2-S7

**Published:** 2013-01-21

**Authors:** Raj K Gaire, Lorey Smith, Patrick Humbert, James Bailey, Peter J Stuckey, Izhak Haviv

**Affiliations:** 1 NICTA, Victoria Laboratory and Department of Computing and Information Systems, University of Melbourne, Parkville, Vic 3010, Australia; 2 Metabolomics, Population Studies and Profiling, Baker IDI Heart and Diabetes Institute, Melbourne, Vic 3004, Australia; 3 Cell Cycle & Cancer Genetics, Peter MacCallum Cancer Centre, Melbourne, Vic 3002, Australia; 4 Department of Pathology, School of Medicine, University of Melbourne, Parkville, Vic 3010, Australia; 5 Faculty of Medicine in Galilee, Bar Ilan University, Israel

## Abstract

Gene expression profiles can show significant changes when genetically diseased cells are compared with non-diseased cells. Biological networks are often used to identify *active subnetworks (ASNs) *of the diseases from the expression profiles to understand the reason behind the observed changes. Current methodologies for discovering ASNs mostly use undirected PPI networks and node centric approaches. This can limit their ability to find the meaningful ASNs when using integrated networks having comprehensive information than the traditional protein-protein interaction networks. Using appropriate scoring functions to assess both genes and their interactions may allow the discovery of better ASNs.

In this paper, we present CASNet, which aims to identify better ASNs using (i) integrated interaction networks (mixed graphs), (ii) directions of regulations of genes, and (iii) combined node and edge scores. We simplify and extend previous methodologies to incorporate edge evaluations and lessen their sensitivity to significance thresholds. We formulate our objective functions using mixed integer programming (MIP) and show that optimal solutions may be obtained.

We compare the ASNs obtained by CASNet and similar other approaches to show that CASNet can often discover more meaningful and stable regulatory ASNs. Our analysis of a breast cancer dataset finds that the positive feedback loops across 7 genes, *AR*, *ESR*1, *MYC*, *E*2*F*2, *PGR*, *BCL*2 and *CCND*1 are conserved across the basal/triple negative subtypes in multiple datasets that could potentially explain the aggressive nature of this cancer subtype. Furthermore, comparison of the basal subtype of breast cancer and the mesenchymal subtype of glioblastoma ASNs shows that an ASN in the vicinity of *IL*6 is conserved across the two subtypes. This result suggests that subtypes of different cancers can show molecular similarities indicating that the therapeutic approaches in different types of cancers may be shared.

## Background

The full genome sequencing of cancer cases demonstrates how remarkably heterogeneous cancer cases are [1]. This heterogeneity is consistent with the hypothesis that most mutations are innocent bystander consequences of the failure of cancer cells' intrinsic mechanism to repair and guard the integrity of the genome [2]. However, the observed heterogeneity of the cancer mutations combined with the knowledge of multiple lesions that all could lead to the same phenotypic consequence [3], leads to a new emerging hypothesis. According to this competing hypothesis, intrinsic subtype specific cancer causing mutations are rare, but their biological output is common [4].

The recognition that *cancer stem cells *within a tumour mass uniquely carry the potential for overt malignancy [5,6] and the discovery that these cells can be transformed into and change forms between epithelial or mesenchymal cells, a phenomena known as epithelial-mesenchymal transformation (EMT) [7], has increased our insight into the link between EMT and fatal cancer phenotypes, such as metastasis and resistance to treatments. In addition, the discovery of intrinsic subtypes of breast cancer that express unique groups of genes [8] has advanced its prognosis. Some intrinsic subtypes of breast cancer are associated with elevated susceptibility to specific drugs, such as Herceptin (for amplified *HER*2 cases) and Tamoxifen (for ER+ cases), while other subtypes, such as the mesenchymal basal/triple negative cases remain without a matching therapeutic strategy. Being able to compare subtypes of different cancers may help identify genes causing the specific subtypes of cancers, leading to identify better therapeutic targets. More importantly, this could provide a scientific basis to sharing therapeutic strategies in subtypes of different cancers.

The task of interpreting gene expression profiles in a disease is not only to differentiate the non-random changes from the random and irrelevant changes, but also to identify the disease causing changes, their downstream effects and the cellular responses related to the disease. If such a procedure worked, one would expect to see the intrinsic signature of luminal breast cancer subtype emerging as downstream to gene *ER*. Furthermore, one could identify driver mutations of the mesenchymal/basal subtype for which therapeutic strategies fail to work. Biological interaction networks contain immense amount of knowledge suitable for such analysis [9]. Finding active subnetworks in diseases is a typical analysis which uses such networks to generate meaningful biological contexts from the differentially expressed genes.

An active subnetwork (ASN) is a subnetwork of a biological interaction network in which the significant nodes obtained from an experiment are connected by edges defined in the network [10]. A methodology for finding ASN was initially proposed by Ideker *et al. *[10]. When coining the idea of ASNs, they established the goal of finding ASNs that could answer questions such as *"What are the signalling and regulatory interactions in control of the observed gene expression changes? How is this control exerted?" *To achieve this, several variations of their work have been proposed [11-17] to analyse differentially expressed genes in diseases using protein-protein interaction (PPI) networks.

PPI networks are undirected networks. Therefore, the ASNs obtained by using these networks can show signalling and regulatory information, but without the directionality of edges, they cannot explicitly show how the controls are exerted. Furthermore, PPI networks have two problems. One, individual interaction networks exhibit little overlap [18], suggesting that a single interaction network might not contain complete information. To overcome this problem, different interaction networks are combined in single integrated interaction network as a mixed graph, containing different types of biological interactions, such as activation, inhibition and post-translational modification. Second, these networks contain both false positive and false negative edges [19], suggesting that the qualities of edges may not be consistent across the network. This problem is solved by computing confidence values of the edges from the sources from which the edge information is obtained from (e.g. number of sources). Alternatively, the values are defined by co-relations of genes in the experimental data [16].

With the integrated networks containing more comprehensive information, one would expect to obtain more informative ASNs by using them with the existing methods. In fact, Deshpande *et al. *[17] used the direction of regulation of genes in multiple species and showed that the identified ASNs are more stable and consistent across multiple species. Similarly, other tools such as IPA (Ingenuity^® ^Systems, http://www.ingenuity.com) show directed edges in their outputs. However, these methods do not use node and edge information together which could produce better ASNs from this type of networks.

We have identified the three issues: node centricity, sensitivity to p-value thresholds and inability to compare ASNs, which limit the existing methods from finding ASNs that could explain how the genetic controls are exerted in diseases (see supp text for details). In addition, the existing ASN finding tools require users to have a copy of the entire network database prior to starting any analysis, which can further affect their usability.

In this paper, we present CASNet (*C*onsistent *A*ctive *S*ub*net*works), our novel methodology that uses (i) an integrated interaction network, STRING [20], (ii) directions of gene regulations, and (iii) combined node and edge evaluations, to find better ASNs. We model the objective function using a mixed integer programming (MIP) model and then solve the model using CPLEX to efficiently discover optimal ASNs. Furthermore, CASNet uses web based APIs to access only relevant parts of the interaction networks and does not require a local copy of the entire database obtained prior to using this tool. We use simulated datasets to show that CASNet can address the above identified limitations. Additionally, we use publicly available datasets to identify and compare ASNs of cancers that provides interesting biological insights. CASNet and supp text of this paper are available at http://www.csse.unimelb.edu.au/~rgaire/CASNet/.

## Results and discussion

In this section, firstly we will show that by adding edge information, our method not only selects interaction edges which are consistent with the experimental results, but also helps reduce the sensitivity to p-value significance threshold. Secondly, we will present our analysis of breast cancer and comparison of ASNs in mesenchymal subtypes of breast and glioblastoma cancers.

### Comparing with node centric approaches

In order to evaluate CASNet, we used simulated networks of varying nodes and compared our results directly with the result obtained from three different methods (i) jActiveModule [10], (ii) heinz [15] and (iii) CEZANNE [16]. jActiveModule is a Cytoscape [21] implementation of Ideker *et al. *ASN finding problems being NP hard, it uses the simulated annealing heuristic approach to obtain optimal solutions. In contrast, heinz is an implementation of Dittrich *et al. *which models this problem as a Prize Collecting Steiner Tree problem and uses Integer Programming techniques to obtain the exact solution. Heinz is also available as a Bioconductor package, which was used for this comparison. Finally, CEZANNE is MATISSE [22] module and uses not only the significance of nodes but also the similarity of nodes as well as an assessment of edges based on the correlations of nodes. These three methods cover a broad range of techniques that are currently available for finding ASNs. By comparing our results with the results obtained from these methods, we can understand the performance of our method against other similar methods. In addition to this, we also compared the results obtained by using our method disregarding the directionality of edges to understand the performance differences attributed to the directionality. These experiments were performed for different network sizes to assess the robustness of our method. At this point, we note that some approaches have been developed that use co-expression [23], co-variance [14] and correlation [16] of genes for creating or assessing edges in the networks. Although useful, these methods may not be applicable when only a list of genes is available.

We used the following parameters of the individual methods: jActiveModule was used with the default parameters to obtain only 1 module. For heinz, a false discovery rate (FDR) of 0.05 was used (more stringent FDR thresholds had poor performance). For two nodes *a *and *b*, CEZANNE required node similarity scores matrix, *sim*[*a*][*b*]. This was calculated from p-values of the nodes, *p_a _*and *p_a_*, as *sim*[*a*][*b*] = *sim*[*b*][*a*] = *min*(*p_a_*, *p_b_*)/*max*(*p_a_, p_b_*) such that the nodes having similar p-values obtain high similarity scores (≈1) while the nodes having different p-values obtain low similarity scores (≈0). A p-value threshold of 0.05 was used for both CEZANNE and CASNet. Precisions (true positive/all classified as positive) and recalls (true positive/all positives) for both nodes and edges were added for each methods for comparisons.

**Figure 1:**
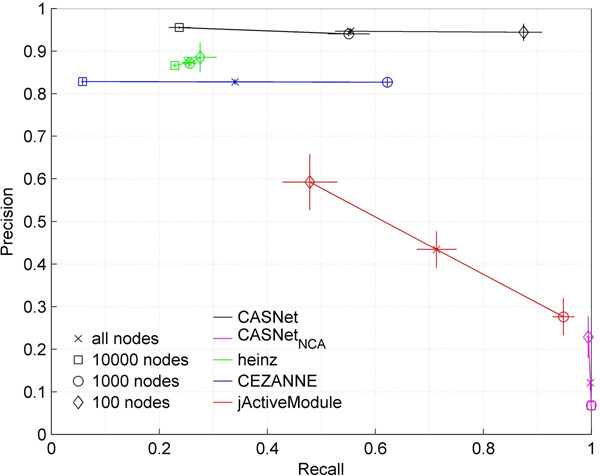
**Comparison of ASN finding methods in simulated datasets**.

Figure 1 illustrates the performance of different methods. It shows that jActiveModule finds modules with low precision and high recall. Therefore, the modules obtained by this method are often bigger and may contain large number of false positives. In contrast, heinz produces modules with high precision but low recall, hence discards many true positive nodes and edges. CEZANNE produced ASNs with better recall, but worse precision than heinz. Note that CEZANNE and jActiveModule failed to find modules in small and large networks respectively. Additionally, jActiveModule produced inconsistent precision and recall whereas CEZANNE produced consistent precision but inconsistent recall for different network sizes. In contrast, heinz performed consistently, though with lower recall rates.

Since CASNet relaxes the significance threshold, it starts by selecting a large number of nodes. The edges connecting these nodes are then assessed based on their directionality and confidence scores. This is also apparent in Figure 1. Here, CASNet without considering directionality of edges (*CASNet_NCA_*) performs with high precision but low recalls. The recall in CASNet is highly improved by using edge information. This demonstrates that by using additional information of edges, the quality of ASNs can be dramatically improved (see Supp text for further comparisons).

### Analysis of cancer datasets

In a recent study, Iliopoulos *et al. *[24] noted that the genes related to inflammatory pathways form stable PFLs regulated by *IL*6 causing continuous progression of cancer. Their study involved extensive laboratory based works. By using computational methods instead, we can potentially not only reduce costs and efforts of identifying PFLs, but also identify novel PFLs.

#### ASNs and PFLs in breast cancer

We used a publicly available breast cancer dataset to explore ASNs and PFLs in the basal subtype of breast cancer. SAM analysis [25] was performed to obtain a list of significant genes. This list was used with STRING network [20] to find ASNs and PFLs.

**Figure 2:**
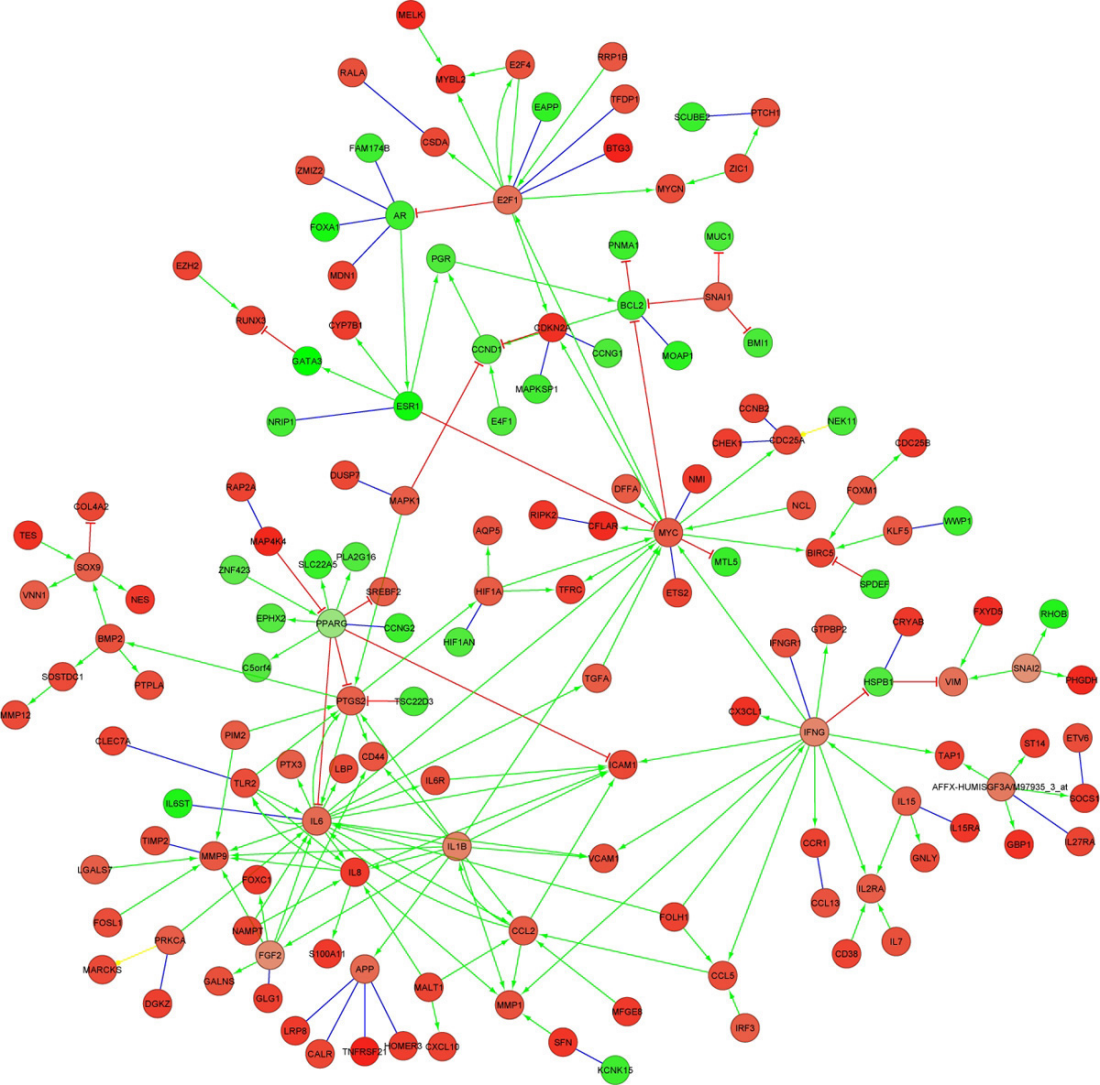
**ASN in ER- subtype of breast cancer**. Here, the red and green circles represent the genes that are respectively over- and under-expressed in ER-cases.

**Figure 3:**
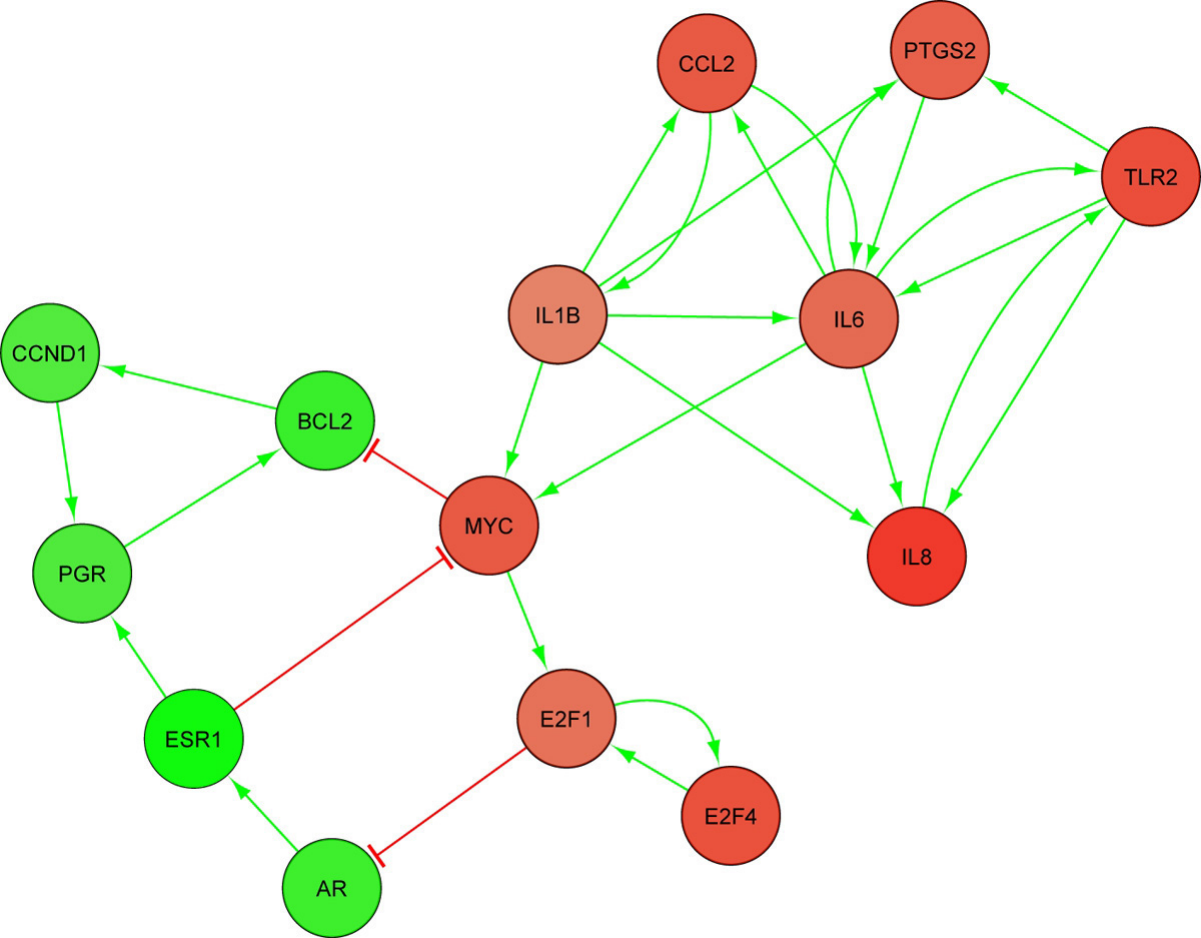
**PFLs in ER- subtype of breast cancer**.

**Figure 4:**
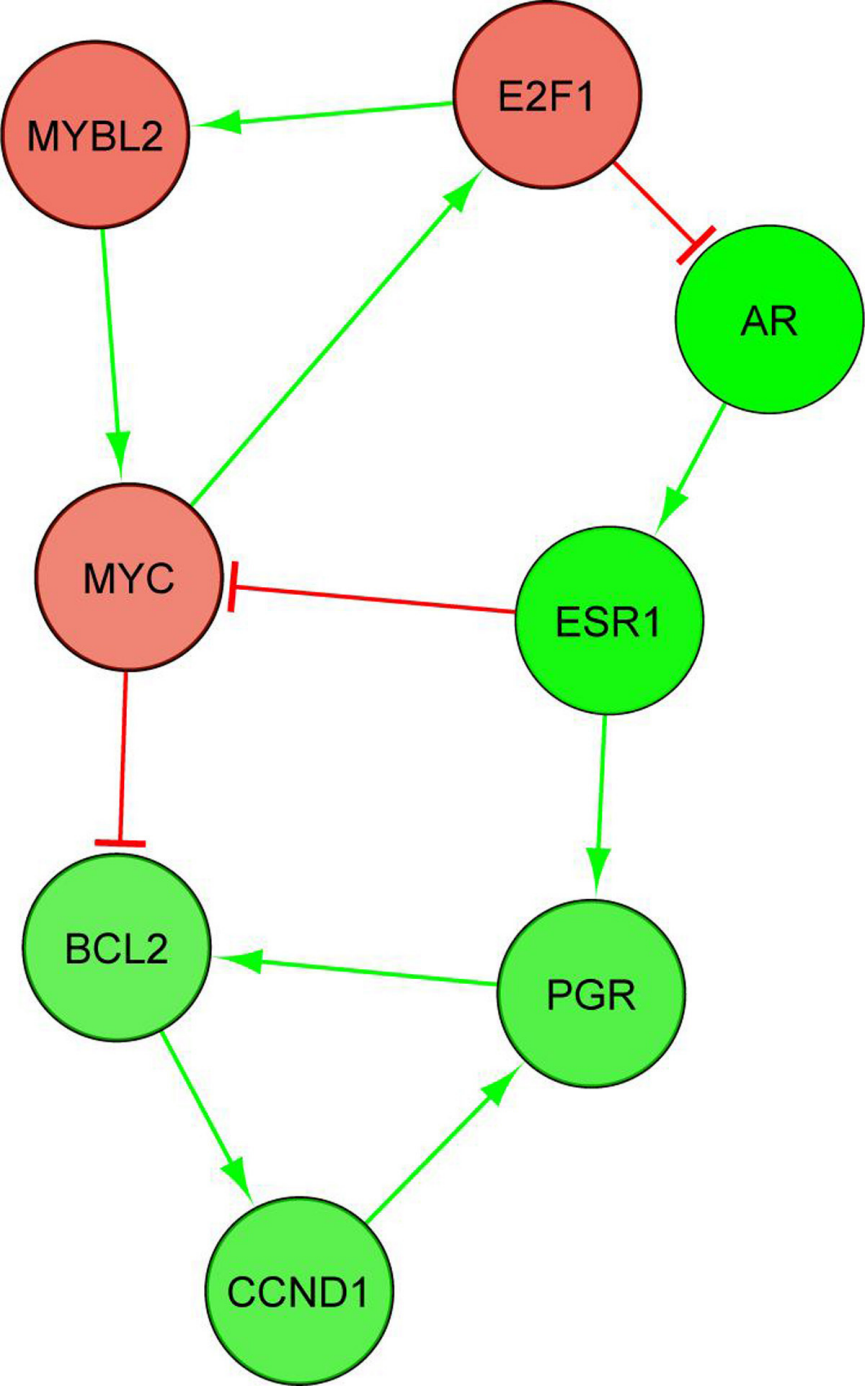
**Conserved PFLs in basal/triple negative subtype of breast cancer in two independent datasets**.

Figure 2 is the ASN obtained from the gene list of basal breast cancer. This ASN contains PFLs as shown in Figure 3. Besides other genes, we found that the PFLs across *MYC*, *E*2*F*1, *AR*, *ESR*1, *CCND*1, *PGR *and *BCL*2 were conserved across independent breast cancer dataset as shown in Figure 4. However, we did not find any PFL when the differentially expressed genes from the entire dataset without discriminating the cancer subtypes were used. *ESR *(i.e. *ER*) is one of the genes which differentiates luminal and basal subtypes [8]. The breast cancer patients with low *ER *expression levels have poorly survival rates. Our identification of these PFL in ER- samples with oncogene like *MYC *and tumour suppressor gene like *CCND*1 with the breast cancer discriminating gene *ESR*1 is a novel finding. Existence of such PFLs probably explains the reasons behind the resistance to the therapeutic in this cancer subtype.

In order to obtain independent evidence for the CASNet choice of expression-based nodes and edges, we used the TCGA query portal [26] to find genes included in the ASN which had consistent expression and copy number changes. Here, we assumed that a gene could be causal if it is not affected by other genes and have mRNA expression level changes consistent with the copy numbers of the genes. *FOXA*1, *NCOA*7 and *DOCK*7 were the top three genes in this result. Since *DOCK*7 is a downstream signalling intermediate of *ERBB*2, identification of *DOCK*7 aberrations in the absence of *HER*2 over-expression may implicate *DOCK*7 in Transtuzumab drug resistance. Moreover, when *FOXA*1 and *NOCA*7 were considered with the PFL forming genes, all the samples had at least one gene that had consistently changed expression levels. This further suggests that PFLs and their neighbouring genes could be important in understanding the nature of complex cancer cases.

#### ASNs in glioblastoma vs breast cancer

Here, we used Glioblastoma (GBM) dataset from Verhaak *et al. *[27] to obtain an ASN for mesenchymal subtype cases and compare it with the ER- breast cancer ASN, since both of these subtypes of cancers show mesenchymal signatures, have poor patient survival and are likely to be related to the EMT event.

**Figure 5:**
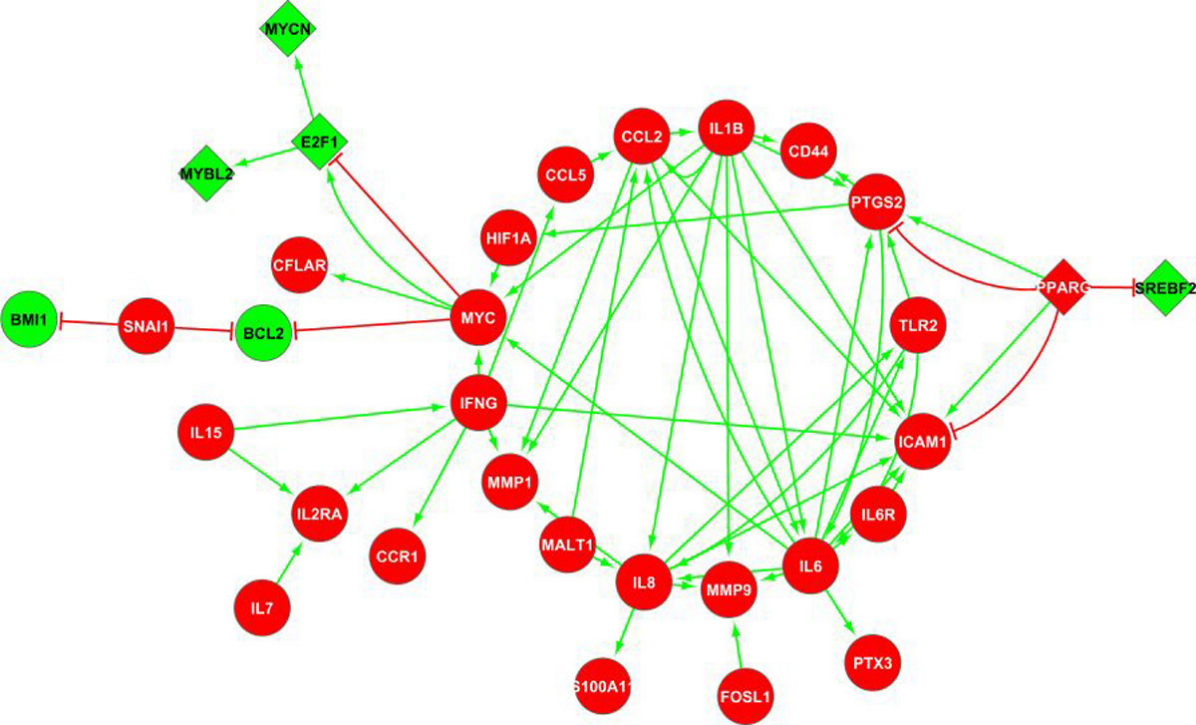
**Conserved subnetwork in mesenchymal glioblastoma and ER- breast cancers**. Here, the red and green circles represent the genes that are respectively over- and under-expressed in the subtypes of both cancers. The red and green diamonds represent the genes which are respectively over-expressed in glioblastoma but low in ER- cases and vise-versa. It shows that not only the inflammation regulating genes such as *IL*6 and *IL*8, but also the mesenchymal marker genes such as *CD*44 and *SNAI*1 are conserved across the two subtypes.

Figure 5 shows the similar components of ASN in these subtypes of cancers. Here, *PPARG*, *SREBF*2, *E*2*F*1, *MYBL*2 and *MYCN *are the genes which are differently regulated while several other genes are similarly regulated in the two cancer subtypes. *PPARG *is up-regulated in mesenchymal GBM, but down-regulated in ER- breast cancer. This gene can both enhance and suppress the expression of *PTGS*2 and *ICAM*1, which are up-regulated in both subtypes. *PTGS*2 (*COX*2, an aspirin target) is a key mediator of inflammation [28]. This behaviour of *PPARG *is likely expressed by stromal adipocytes (fat cells), which are known to accentuate inflammatory processes in a number of human pathologies, including cancer.

*IL*6 is the most connected node in this similarity network. It is associated with acute inflammation, suggesting that higher expression of *IL*6 could be a cellular response to inflammation. *MYC*, which is found to be forming PFLs in basal/triple negative breast cancer cases, is seen to be regulated by *IL*6, *IL*1*B*, *IFNG *and *HIF*1*A*, and is conserved in both mesenchymal GBM and ER- breast cancer. This independently confirming the role of *IL6 *in cancers and suggests that maintaining high level of inflammation may be a conserved feature of mesenchymal subtypes of cancers. More generally, our finding of the common pathways in the different subtypes of cancers suggests that even though the genetic signatures among different cancers may not be similar, the cancer subtypes might not only have similarities in their genetic signatures but also have similarly affected pathways and could potentially be treated in the same manners.

## Materials and methods

In this section, we describe the datasets and our methodology for finding ASNs.

### Simulated networks

Here, we created simulated experimental datasets and consistent gold standard ASNs. These ASNs were combined to obtain simulated network. The experimental datasets and the simulated networks were then used to obtain ASNs from different methods and compared against the gold standard ASNs. More specifically, we fist created nodes of sizes *N* = 100; 1000; 10000. p-values were then assigned to these nodes such that *n* = 0:1 x *N* randomly selected nodes obtained values smaller than 0:001 (considered as the significant nodes), while other nodes were assigned uniformly distributed values between 0 and 1. 2 x *n* random pairs of significant nodes were assigned directed up- and down-regulating edges consistent with the direction of regulation of nodes. The confidence scores of all the edges were assigned a constant value, 1. Each of the above experiment with different node sizes was repeated for 10; 15 and 20 times by randomly reassigning p-values but changing the directions of regulations of a fraction (0:5) of significant nodes to add randomness in the experimental results. Since the edges are reassigned in each experimental data, this variation in node regulation can create different edges among the same two nodes as found in real biological networks.

### Biological network data

We used the action networks of STRING [20] as our network data source. The network was access at run time via the publicly available web based APIs. The benefits of this approach are that downloading and maintaining the entire database is not required, making CASNet usable in a computer with internet connection. The assessed parts of STRING were saved locally for future use, thereby avoiding excessive internet usage.

### Experiment data

The breast cancer samples GSE2034 [29,30] were obtained from obtained from GEO [31]. The GEO dataset contained 282 samples, with 206 were ER+ and 75 ER- cases. The TCGA BRCA dataset contained 465 samples. TCGA [32] (BRCA) dataset was used as an independent validation dataset.

The GBM dataset was obtained from a TCGA publication [27] containing 206 samples. It categorised the samples into 4 subtypes: Proneural (PN), Neural (NL), Classical (CL) and Mesenchymal (MES) based on their genetic signatures. Their SAM analysis [25] results of MES subtype were taken as a basis for the significance measurement of the genes (see supp text for details).

### Interaction networks

Let *V_exp _*be a set of biological molecules being investigated in an experiment. The differential expression analysis finds molecules *M *= (*V_exp_*, *P*, *D*) with p-value significances *P*, and directions of regulations (up- or down-regulation) *D*.

A network (or graph) *G *= (*V*, *E*) consists of vertexes (or nodes) *V *which are connected by edges *E*. Given two nodes *v*_1_, *v*_2 _∈ *V*, and edge *e *∈ *E*; *e *= (*v*_1_, *v*_2_, *t*, *c*) connects the nodes *v*_1 _and *v*_2 _by an edge with a type *t *and a confidence value *c*. The confidence value of an edge is in the range of (0, 1] where 0 is the least confidence and 1 is the most confidence value. Two nodes may be connected by multiple edges with different values of *t*. The following four types of edges (*t*) are defined between nodes: (i) physical binding of two nodes, denoted by *v*_1 _- *v*_2_, (ii) catalytic and post-transcription modification of a node by another node, denoted by *v*_1 _⊸ *v*_2_, (iii) activation of a node by another node, denoted by *v*_1 _→ *v*_2_, and (iv) inhibition of a node by another node, denoted by *v*_1 _⊣ *v*_2_. For an edge *e*, a confidence value *c *is defined. Now, the problem of finding an ASN can be stated as follows:

#### Problem statement

Given an interaction network, *G *= (*V*, *E*), and a differentially expressed (DE) gene dataset, *M *= (*V_exp_*, *P*, *D*), find the best (where the notion of best is defined by scoring functions) subnetwork *G*' = (*V'*, E') ⊂ *G *which connects the highly DE genes *v *∈ *V'*, *V_exp _*⊂ *V *with edges *E' *⊂ *E *that are consistent with the data.

#### Consistent edge

An edge *e *= (*n*_1_, *n*_2_, *t*, c) ∈ *E *between two nodes *n*_1_, *n*_2 _∈ *V *is considered to be consistent if the direction of regulation of gene *n*_2 _can be explained by the direction of regulation of *n*_1 _by using the edge type *t *with a high confidence *c*.

For example, if *n*_1 _promotes *n*_2_, i.e. *n*_1 _→ *n*_2_, and both *n*_1 _and *n*_2 _are up- or down-regulated, then the edge *e *= (*n*_1_, *n*_2_, *→*, *c*) is considered to be consistent with the data with a confidence *c*. In contrast, if a node *n*_1 _is up-regulated and the other node *n*_2 _is down-regulated and vice-versa, then the edge is considered to be inconsistent with the data.

Based on the problem statement, we now define the node, edge and subnetwork scores that will be used to evaluate ASNs.

### Node scores

In an experiment, DE genes are often associated with p-values obtained from some statistical tests. A subnetwork with nodes having low p-values is a desirable feature of an ASN [10]. Additionally, it is often desirable to exclude highly connected nodes in an ASN [33-35]. Here we adopt Dittrich *et al*'s [15] node scoring method to include low p-values in the networks (profit). At the same time, we use a simple approach to penalise highly connected nodes (cost).

Let *p_n _*be the p-value of a node *n*. Then the node's profit score *W_n _*is given as [15],

(1) $$W_{n}=\left(a-1\right)\times \left(log\left(p_{n}\right)-log\left(\tau \right)\right)$$

where *a *= (0, 1] is a shape parameter of the beta distribution fitted for a dataset representing a signal to noise ratio, and *τ *is a threshold to control the size of the ASN (interpreted as the false discovery rate). In this case, the value of (*a *- 1) acts as a scaling factor. This factor is useful to compare the scores of a node obtained from different datasets having different p-value distributions. On the other hand, *τ *can be a selected threshold which can make a node score positive or negative, and thereby controls the size of the ASNs. Since the value of *a *does not affect the sign of the scores, and if a single p-value dataset is used, *a *can be assigned a constant value without affecting the resulting ASN. Practically, *a *is close to 0. Therefore, the node score can be simplified by assigning *a *= 0 as *W_n _*= -1 × (*log*(*p_n_*) - *log*(*τ*)). Furthermore, if a set of significant genes is used in an experiment after applying a p-value threshold, the Eq. 1 can be further reduced to, *W_n _*= 1 × *log*(*p_n_*) for all the genes in the set and *W_n _*= -1 × |*Constant*| for the genes not in the list.

If *D *is the degree of a node *n *(i.e. it is connected to *D *other nodes), we assign a cost *C_n _*to the node to penalise highly connected nodes, as, *C_n _*= *log*(*D*).

Since the profit and the cost scores of a node do not have the same scale, we scale these values to a range of [-1, 1] to obtain *standard *scores as, $W_{n}^{\prime }=\frac{W_{n}}{max_{m\in V}\left(\left|W_{m}\right|\right)};C_{n}^{\prime }=\frac{C_{n}}{max_{m\in V}\left(\left|C_{m}\right|\right)}$

### Edge scores

It is desirable to assign a high *positive *score to an edge which is consistent with the data, so that including such edges will increase the subnetwork scores. Similarly, the inconsistent edges should be penalised by assigning *negative *scores to them. Additionally, the confidence value of an edge should be used to scale these scores. Therefore, we use the following scheme to obtain the edge scores:

1. If *n*_1 _promotes *n*_2_, then the consistency score *W_e _*is equal to (i) 2 if both *n*_1 _and *n*_2 _are changed in the same direction, (ii) -1 if either *n*_1 _or *n*_2 _is unchanged and (iii) -2, if *n*_1 _and *n*_2 _are changed in opposite direction.

2. Similarly, if *n*_1 _suppresses *n*_2_, then the consistency score *W_e _*is equal to (i) 2 if *n*_1 _and *n*_2 _are changed in an opposite direction, (ii) -1 if only one of *n*_1 _and *n*_2 _is changed and (iii) -2, if *n*_1 _and *n*_2 _are changed in the same direction.

3. For edges *n*_1 _⊸ *n*_2 _and *n*_1 _- *n*_2_, a constant value can be assigned depending on whether including such edges is desirable or not. For example, *W_e _*= 1 could be used in PPI networks, while *W_e _*= -1 could be used in the networks where having these edges is not desirable. By default, we use *W_e _*= -1 to lessen emphasis on undirected edges.

Now, an edge score is defined as, *S_e _*= *W_e _*× *C_e _*where *C_e _*= (0, 1] is the confidence score of the edge obtained from the interaction network.

Finally, the *standard *edge score is obtained as, $S_{e}^{\prime }=\frac{S_{e}}{max_{f\in E}|S_{f}|}$

### Sub-network score

Based on the standard node and edge scores, the score of a subnetwork *G*'-is obtained by-using the linear equation,

(2) $$S=\underset{n\in V}{\sum }x_{n}\times \left(\alpha \times {W^{\prime }}_{n}-\beta_{n}\times C_{n}^{\prime }\right)+\gamma \;\times \underset{e\in E}{\sum }x_{e}\times {S^{\prime }}_{e}$$

where *α*, *β *and *γ *are the scaling factors of the node weight profits, the node connectivity costs and the edge consistency scores respectively and *x_n _*and *x_e _*are boolean variables with values 1 if *n *∈ *V'*, *e *∈ *E*' and 0 otherwise. The values of these scaling factors could be obtained by using gold standard network and experiment datasets. In the absence of such datasets, we use *α *= *γ *= 1 and *β *= 0 for the *positive *scoring nodes and *β *= 1 for *negative *scoring nodes in our experiments. This is because a large number of edges around cancer related genes exist in the biological networks, since a high number experiments have been performed in those genes. Penalising them at the same rate as others eliminates the highly DE genes (results not shown).

The objective function for finding ASNs is to obtain a sub-network which maximises the subnetwork score *S*.

### The MIP model

Here, we model the problem of finding an ASN by using the mixed integer linear programming (MIP) model in CPLEX which maximises the objective function in Eq. 2. *x_n _*and *x_e _*are defined as boolean variables (i.e. *x *∈ 0, 1). Further to this, the following additional constraints are imposed: (a) *x*_*n*(*i*) _→ ∃*x*_*e*(*i, j*) _i.e. *x*_*n*(*i*) _≤ Σ*_j_*x_*e*(*i, j*)_. (b) *x*_*e*(*i, j*) _→ *x*_*n*(*i*)_; i.e. *x*_*e*(*i, j*) _≤ *x*_*n*(*i*)_. (c) *x*_*e*(*i, j*) _→ *x*_*n*(*i*)_; i.e. *x*_*e*(*i, j*) _≤ *x*_*n*(*i*)_. where *n*(*i*) is the *i^th ^*node and *e*(*i*, *j*) is an edge connecting the nodes *n*(*i*) and *n*(*j*).

## Conclusion

A large number of datasets that are currently being produced, such as TCGA and ICGC, include definitive genome wide mutational status of many samples, making the task of interpreting the results, and identifying common features even more formidable. Adapting to these high dimensional datasets, biological interaction databases are being integrated into single databases to provide more comprehensive information. Since all the interactions among the nodes might not be active at the same point of time or environmental conditions, the current methodologies of enrichment assessment for candidate networks or pathways can fall short in their ability to discriminate between real biological inferences from false positive ones. Better methodologies are required to use these networks and systematically find interesting and meaningful interactions in disease conditions.

In this paper, we presented a methodology to analyse gene expression results of diseases with STRING network, to not only find a connected subset of nodes that are observed to be highly differentially expressed, but also use the edges in the network to generate hypotheses regarding the reason behind the observed changes. Our methodology, CASNet, enhances existing methodologies by introducing edge scores to solve node centricity, p-value sensitivity problems and subnetwork comparability problems.

We demonstrated that complicated regulatory ASNs and PFLs exist in low surviving cancer cases such as the mesenchymal subtype of GBM and the basal/triple negative subtype of breast cancer. Finally, we showed that by comparing ASNs of different disease types, molecular similarities of the disease can be identified that can be useful in their treatments. In this way, CASNet has widened the possibilities of network analysis in generating biologically significant hypotheses and directing the future researches.

### Limitations

Firstly, the current state of directed interaction network has low coverage. Additionally, a large number of edges around the genes of well studied diseases, including cancer, exist due to the large number of experiments that have been carried out with those genes. This creates a bias toward some parts of networks. The parts of network where directionality information exists are most likely the pathways that are already well-understood. As such, the discovery of new genes and their interactions from these networks may be less likely. Secondly, we found several instances where the edges are incorrectly defined in STRING. The edges in our ASNs are only as good as the curation and literature mining methodologies used in creating the networks. Finally, the interaction networks do not differentiate wild type/mutant and active/inactive molecules in their nodes. In the absence of this level of sensitivity in the existing biological networks, precise conclusions cannot be made. Consequently, the results obtained from ASN finding methods are susceptible to the problems associated with the underlying networks and the datasets being used, and hence require independent validations.

## Competing interests

The authors declare that they have no competing interests.

## Authors' contributions

RKG, JB, PJS, IH - conception, design, development, data acquisition, analysis, interpretation of results, draft manuscript; LS, PH - conception, preliminary interpretation of results.

## Declaration

The publication costs for this article were funded by the University of Melbourne.

This article has been published as part of *BMC Bioinformatics *Volume 14 Supplement 2, 2013: Selected articles from the Eleventh Asia Pacific Bioinformatics Conference (APBC 2013): Bioinformatics. The full contents of the supplement are available online at http://www.biomedcentral.com/bmcbioinformatics/supplements/14/S2.
